# Assessment and hydro-geochemical characterization for evaluation of corrosion and scaling potential of groundwater in South West Delhi, India

**DOI:** 10.1016/j.dib.2018.03.120

**Published:** 2018-03-31

**Authors:** Sanigdha Acharya, S.K. Sharma, Vinita Khandegar

**Affiliations:** University School of Chemical Technology, Guru Gobind Singh Indraprastha University, Dwarka, New Delhi, India

**Keywords:** Groundwater, Hydro-geochemical, Corrosion, Scaling, Water stability index, Delhi

## Abstract

In the present study, hydro-geochemical characteristics of groundwater samples collected from South West Delhi, India, have been assessed. 50 sampling locations were recorded with the help of global positioning system, to assess the groundwater quality and evaluate the corrosion and scaling potential. Hydro-geochemical characterization for different parameters such as pH, temperature (T), electrical conductivity (EC), total dissolved solids (TDS), salinity (SA), total hardness (TH), total alkalinity ($HCO_{3}^{-}$), levels of anions such as calcium (Ca^+2^), magnesium (Mg^+2^), sodium (Na^+^), potassium (K^+^) and cations which include chloride (Cl^-^), Flouride (F^-^), sulfates ($SO_{4}^{-2}$), Nitrates ($N{O_{3}}^{-}$) was done using standard APHA methods. The corrosion and scaling potential of groundwater was evaluated by five stability indices: Langelier saturation index (LSI), Ryznar stability index (RSI), Aggressive index (AI), Learson–Skold index (Ls) and Puckorius scaling index (PSI). The dataset classified groundwater as polluted and this indicates that the water is not safe for domestic, agricultural and industrial usage and will need further treatment. This dataset is beneficial for policymakers, and researchers in the field of water purification, quality management and in preventing the economic and safety concerns related to corrosion and scaling of groundwater.

**Specifications Table**

**Table:** 

Subject area	Environmental Science
More specific subject area	Groundwater study
Type of data	Table and Figure
How data was acquired	Water analysis kit via NPC363D, India, UV–vis Double Beam spectrophotometer (Hitachi U-2900, India), Flame photometer (Toshniwal TMF-45, India).
Data format	Raw, analyzed
Experimental factor	Groundwater samples from 50 different areas of South-West Delhi, India were collected from sources such as bore-wells, private and government hand pumps in the year 2016–17. All sampling sites were selected with a view to cover the entire area of the study area.
Experimental features	Determination of hydro-geochemical parameters that represent the contamination of the studied groundwater samples.
Data source location	South-West Delhi, New Delhi, India
Data accessibility	This article contains corrosion and scaling potential dataset.

**Value of the data**

• Determination of hydro-geochemical characterization such, T (°C), EC (µS/cm), pH TDS, SA, TH, $HCO_{3}^{-}$, Ca^+2^, Mg^+2^, Na^+^, K^+^ , F^−^, Cl^−^, $SO_{4}^{-2}$ and $N{O_{3}}^{-}$ (all values in mg/L except pH) in 50 groundwater samples of South West Delhi, India.
• The chemistry of groundwater is an important factor determining its use for domestic, irrigation and industrial purposes. Due to limited literature available for the study area, this data can help to better understand the quality of groundwater and provide information for further studies in the field of purification and better water quality management.
• The occurrence of scaling and corrosion may create staining and blocking of piping systems leading to economic and safety problems. In addition, corrosion products contaminate the water resulting in health problems. Therefore, corrosion control is an important aspect of safe drinking water supplies.
• Corrosion indices calculated are important in the monitoring of water supply distribution networks.

## 1 Data

**Fig. 1:**
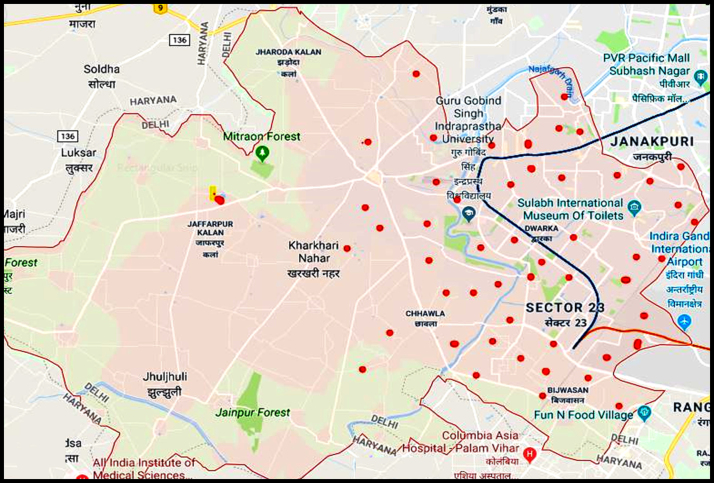
Sampling points of the study area.

**Fig. 2:**
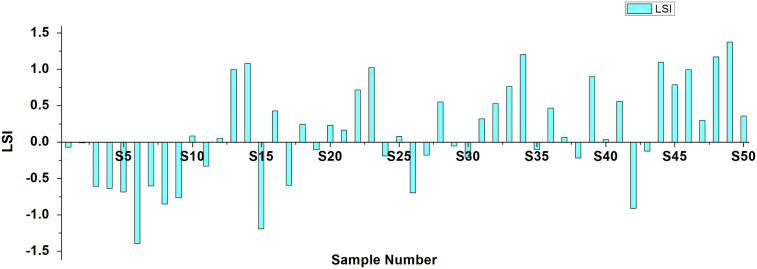
Langelier saturation index.

**Fig. 3:**
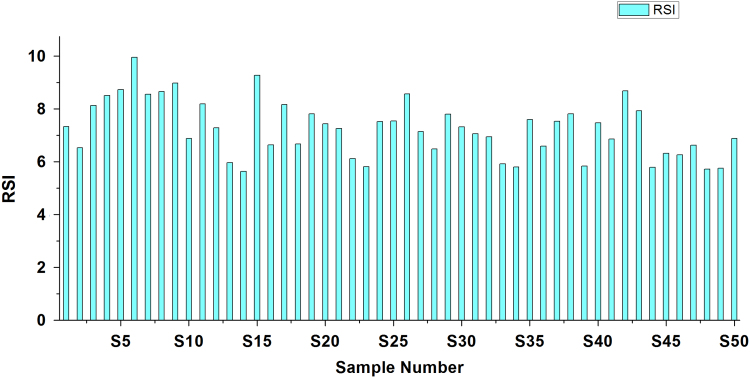
Ryznar stability index.

**Fig. 4:**
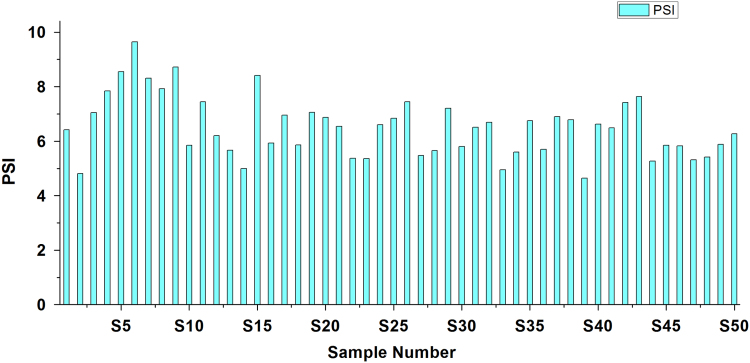
Aggressive index.

**Fig. 5:**
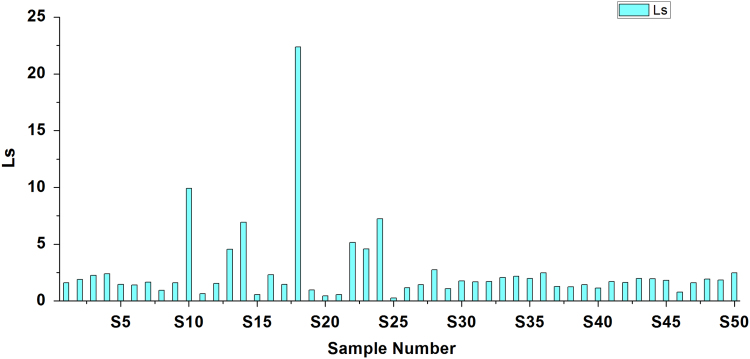
Learson–Skold index.

**Fig. 6:**
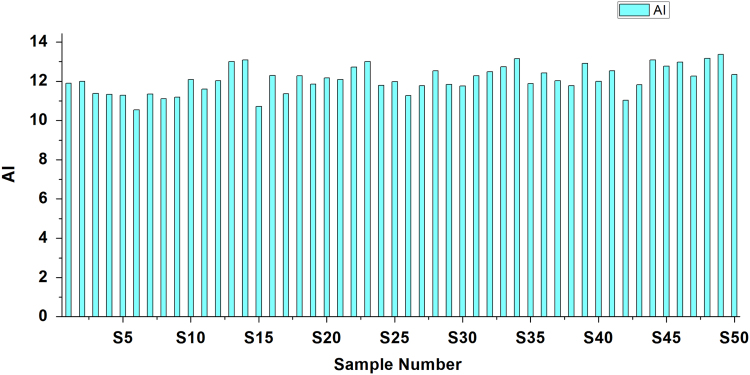
Puckorius scaling index.

This dataset contains 5 Tables and 6 Figures that represent quality as well as corrosion and scaling potential of groundwater of the South West Delhi, India. Fig. 1 shows the sampling points of the study area. Table 1 shows the hydro-geochemical characterization including pH, T, EC, TDS, SA, TH, $HCO_{3}^{-}$ , Ca^+2^, Mg^+2^, Na^+^, K^+^, Cl^−^, F^−^, $SO_{4}^{-2}$, $N{O_{3}}^{-}$ determined using APHA method [1]. The statistical parameters (minimum, maximum, mean and standard deviation) of hydro-geochemical parameters and limits prescribed by World health organization (WHO [2]) and Bureau of Indian Standards (BIS [3]) are tabulated in Table 2 [2], [3]. Criteria and summary of water stability indices are shown in Tables 3 and 4, respectively. Table 5 shows the result obtained by LSI, RSI, PSI, LS, AI analysis of the studied area (Fig. 2, Fig. 3, Fig. 4, Fig. 5, Fig. 6).

**Table 1:** Hydro-geochemical characterization of 50 groundwater samples of South West Delhi, India.

**Sample numbers**	**Temp (°C)**	**pH**	**EC (µS/cm)**	**TDS (mg/L)**	**Salinity (mg/L)**	**Hardness (mg/L)**	**Sodium (mg/L)**	**Potassium (mg/L)**	**Calcium (mg/L)**	**Magnesium (mg/L)**	**Nitrate (mg/L)**	**Fluoride (mg/L)**	**Sulphate (mg/L)**	**Chloride (mg/L)**	**Alkalinity (mg/L)**
S1	27.5	7.18	5070	2535	4910	1336	514	8	201.8	200.13	1.32	1.2	100	321.5	**265**
S2	27.5	6.51	11,290	5645	10,190	4310	650	28	943.9	418.38	0.122	1	120	501.5	330
S3	26	6.92	3450	1725	2415	487	334	12	124.67	45.8	0.135	0.72	71	456.7	234
S4	27	7.24	2600	1300	1820	480	256	3	60.9	40.9	1.67	0.44	59	421	200
S5	26.5	7.35	2460	1230	1722	510	200	19	77.5	61.5	3.9	0.65	47	112.2	110
S6	27.5	7.16	1600	800	1120	150	139	7	23.8	20.7	2.9	0.78	38	100.9	100
S7	26.9	7.35	1800	900	1260	510	154	11	81.7	56.5	0.622	0.35	44	154.7	121
S8	27	6.96	2389	1194.5	1672.3	966	187	10	98	39.9	8.98	0.67	56	76.8	143
S9	27.5	7.45	3406	1703	2384.2	146	200	11	39.08	19.52	1.136	0.57	71	160.4	144
S10	27.5	7.05	8030	4015	5621	2141.4	245	6	410.58	242.87	1.896	0.76	110	2506.9	264
S11	27.7	7.52	1600	800	1120	206	178	6	34.48	35.81	21.194	0.98	42	176.2	345
S12	27.8	7.39	4320	2160	3024	526	213	11	89.19	59.22	0.234	0.57	98	638.4	480
S13	27.1	7.96	7540	3770	5278	2078	230	13	320.64	301.94	0.023	1.9	132	1439.5	345
S14	27.1	7.8	6500	3250	4550	2306	213	6	416.83	336.29	0.647	1.5	128	3061.45	460
S15	27	6.89	726	363	508.2	124	27	7	43.06	22.18	43	1.1	0	87.53	155
S16	27.1	7.49	340	170	238	860	24	8	204.37	106.92	1.818	0.33	0	728.4	315
S17	27	6.98	2680	1340	1876	484	120	11	77.53	75.94	2.878	0.62	87	375.8	315
S18	27.3	7.16	15,780	7890	11,046	3738	780	35	598.2	520.98	2.197	1.9	240	4678	220
S19	27.3	7.6	2690	1345	1883	167.32	165	12	44.05	28.11	1.214	0.9	89	306.63	405
S20	27.3	7.89	1658	829	1160.6	183	49	9	41.06	22.16	0.338	0.34	0	203.8	460
S21	27.3	7.58	1130	565	791	403	23	7	88.26	32.52	0.139	0.5	7	189.05	360
S22	27.3	7.55	8720	4360	6104	2203	197	27	402.75	336.39	2.945	0.7	120	1785	370
S23	27.2	7.87	5220	2610	3654	1803	164	12	361.75	219.83	2.308	0.7	108	1652.5	385
S24	27.3	7.14	5500	2750	3850	1146.66	123	14	182.46	132.12	0.538	1	112	1692.8	250
S25	27.3	7.69	659	329.5	461.3	120	23	13	45.06	23.52	0.195	0.4	23	90.3	425
S26	27.2	7.17	2867	1433.5	2006.9	180	54	11	34.05	30.11	20.75	0.9	39	387.1	365
S27	27.3	6.78	2230	1115	1561	813.33	45	7	212.38	96.49	1.271	0.8	22	637.4	460
S28	27.2	7.59	4970	2485	3479	1146	153	13	198.76	176.41	1.412	0.6	47	1180.8	445
S29	27	7.69	518	259	362.6	170.67	23	1	39.06	22.1	0.549	0.56	32	356	360
S30	27.5	6.9	4659	2329.5	3261.3	1054	200	11	165.45	81.7	27.6	0.67	106	678.97	445
S31	27.5	7.7	2870	1435	2009	991	69	8	108.98	41.75	1.62	0.45	23	556.9	345
S32	27.5	8	2560	1280	1792	980	58	7	89	42.89	0.686	0.78	45	539.97	340
S33	27.5	7.45	4987	2493.5	3490.9	2156	184	16	445.67	328	3.86	0.6	78	835.98	443
S34	27.5	8.2	2831	1415.5	1981.7	1105	89	9	208.76	109.25	18.84	0.8	63	880.8	434
S35	27.7	7.4	6720	3360	4704	872	260	21	89.19	42.5	21.38	0.45	134	545.61	345
S36	27.8	7.52	4130	2065	2891	1083	179	14	187.88	89.64	25.28	0	257	809.9	434
S37	27.1	7.67	2600	1300	1820	428	47	9	60.6	36.26	1.97	0	167	313.95	375.6
S38	25.1	7.38	2500	1250	1750	456	156	10	57.5	28.76	14.14	0.12	234	309.87	435.5
S39	26.4	7.65	6540	3270	4578	1884	356	23	209.5	157.38	23.98	0.56	345	910.18	880.75
S40	27.1	7.54	2610	1305	1827	458	162	12	68.34	32.15	20.47	0.3	53	420	417.67
S41	27.4	7.98	4680	2340	3276	762	175	14	88.97	59.76	12.79	0.3	200	489	405.56
S42	27.5	6.86	2430	1215	1701	418	120	10	54.5	23.45	1.214	0.56	78	367.17	276.45
S43	27.1	7.68	1718	859	1202.6	457	79	11	62.9	31.78	30.38	0.89	65	367	218.32
S44	27.2	7.98	5554	2777	3887.8	1732	230	17	252.55	186.6	34.39	0.56	268	719.67	506.45
S45	27.3	7.9	4620	2310	3234	1246	175	13	176.69	120.66	28.45	0.76	32	726.9	418.34
S46	27.2	8.25	4540	2270	3178	737	158	14	78.8	68.76	18.37	0.7	46	467.87	674.56
S47	27.1	7.22	3383	1691.5	2368.1	1401	100	10	209.16	145.35	32.84	0.8	54	801.98	534.54
S48	27.5	8.06	6978	3489	4884.6	2064	198	26	310.35	287.95	23.28	0.9	67	719.5	410.56
S49	27.3	8.5	5670	2835	3969	1592	210	18	178.09	146.87	15.8	0.46	118	639.98	410.75
S50	27.5	7.6	4925	2462.5	3447.5	1537	154	11	168.56	120.55	13.6	0.56	135	665.76	323.15

**Table 2:** Statistics of hydro-geochemical parameters with limits prescribed by (WHO and BIS) [2], [3].

**Parameter**	**Mean**	**Min**	**Max**	**St.dev**	**WHO standards**	**BIS standards**
Temp (**°**C)	27.21	25.1	27.8	0.4	–	–
pH	7.49	6.51	8.5	2849.2	6.5–8.5	6.5–8.5
EC (µs/cm)	4104.96	340	15,780	1424.6	1500	–
TDS (mg/L)	2052.48	170	7890	2150.3	500	500
Salinity (mg/L)	2946.43	238	11,046	895.3	–	–
Hardness (mg/L)	1062.15	120	4310	895.6	450	300
Sodium (mg/L)	180.84	23	780	145.1	200	200
Potassium (mg/L)	12.44	1	35	6.5	12	–
Calcium (mg/L)	175.35	23.8	943.9	170.9	75	75
Magnesium (mg/L)	118.15	19.52	520.98	117.6	50	30
Nitrate (mg/L)	9.95	0.023	43	11.8	45	45
Fluoride (mg/L)	0.69	0	1.9	0.4	1.5	1
Sulphate (mg/L)	92.20	0	345	74.7	250	200
Chlorides (mg/L)	744.92	76.8	4678	824.6	250	250
Alkalinity (mg/L)	358.10	100	880.75	141.0	500	200

**Table 3:** Corrosion and scaling index criteria [4], [5], [6], [7], [8], [9], [10], [11].

Index	Equation	Index value	Condition
Langelier Saturation Index (LSI)	LSI=pH−pH_s_	LSI<0	No potential to scale, water will dissolve CaCO_3_
	pH_s_=(9.3+A+B)−(C+D)	LSI>0	Scale can form, CaCO_3_ precipitation may occur
	where:	LSI=0	Borderline scale potential
	A=(Log_10_[TDS]−1)/10		
	B=−13.12×Log_10_(°C+273)+34.55		
	C=Log_10_[Ca^2+^ as CaCO_3_]−0.4		
	D=Log_10_[alkalinity as CaCO_3_]		
Ryznar Stability Index (RSI)	RSI=2(pH_s_)−pH	RSI<6	Scale tendency increases as the index decreases
		RSI>7	Calcium carbonate formation probably does not lead to a protective corrosion inhibitor film
		RSI>8	Mild steel corrosion becomes an increasing problem.
Puckorius Scaling Index (PSI)	PSI=2 (pH_s_)−pH_eq_	PSI<6	Scaling is unlikely to occur
	Where:	PSI>7	Likely to dissolve scale
	pH_eq_=1.465×Log_10_[Alkalinity]+4.54		
	Alkalinity= [$HCO_{3}^{-}$ ]+2 [$C{O_{3}}^{2-}$ ]+[OH^−^]		
Larson-skold index(Ls)	Ls=(Cl^–^+$SO_{4}^{-2}$)/($HCO_{3}^{-}$+$C{O_{3}}^{2-}$)	Ls<0.8	Chlorides and sulfate unlikely to interfere with natural film formation
		0.8<Ls<1.2	Chlorides and sulfates may interfere with natural film formation.
		Ls >1.2	High local corrosion tendency expected as the index increases
Aggressive index(AI)	AI=pH+Log_10_[(Alkalinity)(H)]	AI>12	Non aggressive
	Where H=Calcium hardness mg/L	10<AI<12	Moderately aggressive
		AI<10	Very aggressive

**Table 4:** Summary of water stability indices in the present study.

**Sample numbers**	**LSI**	**RSI**	**PSI**	**Ls**	**AI**
S1	−0.07	7.33	6.42	1.59	11.91
S2	−0.01	6.53	4.82	1.88	12.00
S3	−0.61	8.14	7.04	2.26	11.38
S4	−0.64	8.51	7.84	2.40	11.33
S5	−0.69	8.73	8.55	1.45	11.28
S6	−1.39	9.95	9.64	1.39	10.54
S7	−0.60	8.55	8.31	1.64	11.35
S8	−0.85	8.66	7.93	0.93	11.11
S9	−0.76	8.98	8.72	1.61	11.20
S10	0.08	6.88	5.84	9.91	12.09
S11	−0.33	8.18	7.45	0.63	11.60
S12	0.05	7.28	6.21	1.53	12.02
S13	1.00	5.96	5.67	4.56	13.00
S14	1.08	5.63	4.99	6.93	13.08
S15	−1.19	9.27	8.41	0.56	10.71
S16	0.43	6.64	5.93	2.31	12.30
S17	−0.60	8.17	6.95	1.47	11.37
S18	0.25	6.67	5.86	22.35	12.28
S19	−0.11	7.81	7.05	0.98	11.85
S20	0.23	7.43	6.88	0.44	12.17
S21	0.16	7.26	6.55	0.54	12.08
S22	0.71	6.12	5.37	5.15	12.72
S23	1.03	5.82	5.36	4.57	13.01
S24	−0.19	7.52	6.61	7.22	11.80
S25	0.08	7.54	6.84	0.27	11.97
S26	−0.70	8.57	7.44	1.17	11.26
S27	−0.18	7.14	5.48	1.43	11.77
S28	0.55	6.49	5.66	2.76	12.54
S29	−0.05	7.80	7.20	1.08	11.84
S30	−0.21	7.32	5.80	1.76	11.77
S31	0.32	7.06	6.50	1.68	12.28
S32	0.53	6.94	6.69	1.72	12.48
S33	0.77	5.92	4.95	2.06	12.75
S34	1.20	5.80	5.59	2.17	13.16
S35	−0.10	7.60	6.74	1.97	11.89
S36	0.46	6.59	5.71	2.46	12.43
S37	0.07	7.53	6.89	1.28	12.03
S38	−0.22	7.81	6.79	1.25	11.78
S39	0.90	5.84	4.64	1.43	12.92
S40	0.04	7.47	6.63	1.13	12.00
S41	0.56	6.86	6.48	1.70	12.54
S42	−0.91	8.68	7.43	1.61	11.04
S43	−0.12	7.93	7.64	1.98	11.82
S44	1.10	5.79	5.27	1.95	13.09
S45	0.79	6.32	5.84	1.81	12.77
S46	0.99	6.26	5.83	0.76	12.98
S47	0.30	6.63	5.31	1.60	12.27
S48	1.17	5.72	5.41	1.92	13.17
S49	1.37	5.75	5.88	1.85	13.36
S50	0.36	6.89	6.27	2.48	12.34

**Table 5:** Corrosion and scaling potential in the present study.

**Sample numbers**	**LSI**	**RSI**	**PSI**	**Ls**	**AI**
S1	Corrosive	Corrosive	Neutral	High corrosive	Mildly aggressive
S2	Corrosive	Neutral	Scaling	High corrosive	Non aggressive
S3	Corrosive	Corrosive	Corrosive	High corrosive	Mildly aggressive
S4	Corrosive	Corrosive	Corrosive	High corrosive	Mildly aggressive
S5	Corrosive	Corrosive	Corrosive	High corrosive	Mildly aggressive
S6	Corrosive	Corrosive	Corrosive	High corrosive	Mildly aggressive
S7	Corrosive	Corrosive	Corrosive	High corrosive	Mildly aggressive
S8	Corrosive	Corrosive	Corrosive	Corrosion may occur	Mildly aggressive
S9	Corrosive	Corrosive	Corrosive	High corrosive	Mildly aggressive
S10	Scaling	Neutral	Scaling	High corrosive	Non aggressive
S11	Corrosive	Corrosive	Corrosive	Non corrosive	Mildly aggressive
S12	Scaling	Corrosive	Neutral	High corrosive	Non aggressive
S13	Scaling	Scaling	Scaling	High corrosive	Non aggressive
S14	Scaling	Scaling	Scaling	High corrosive	Non aggressive
S15	Corrosive	Corrosive	Corrosive	Non corrosive	Mildly aggressive
S16	Scaling	Neutral	Scaling	High corrosive	Non aggressive
S17	Corrosive	Corrosive	Neutral	High corrosive	Mildly aggressive
S18	Scaling	Neutral	Scaling	High corrosive	Non aggressive
S19	Corrosive	Corrosive	Corrosive	Corrosion may occur	Mildly aggressive
S20	Scaling	Corrosive	Neutral	Non corrosive	Non aggressive
S21	Scaling	Corrosive	Neutral	Non corrosive	Non aggressive
S22	Scaling	Neutral	Scaling	High corrosive	Non aggressive
S23	Scaling	Scaling	Scaling	High corrosive	Non aggressive
S24	Corrosive	Corrosive	Neutral	High corrosive	Mildly aggressive
S25	Scaling	Corrosive	Neutral	Non corrosive	Mildly aggressive
S26	Corrosive	Corrosive	Corrosive	Corrosion may occur	Mildly aggressive
S27	Corrosive	Corrosive	Scaling	High corrosive	Mildly aggressive
S28	Scaling	Neutral	Scaling	High corrosive	Non aggressive
S29	Corrosive	Corrosive	Corrosive	Corrosion may occur	Mildly aggressive
S30	Corrosive	Corrosive	Scaling	High corrosive	Mildly aggressive
S31	Scaling	Corrosive	Neutral	High corrosive	Non aggressive
S32	Scaling	Neutral	Neutral	High corrosive	Non aggressive
S33	Scaling	Scaling	Scaling	High corrosive	Non aggressive
S34	Scaling	Scaling	Scaling	High corrosive	Non aggressive
S35	Corrosive	Corrosive	Neutral	High corrosive	Mildly aggressive
S36	Scaling	Neutral	Scaling	High corrosive	Non aggressive
S37	Scaling	Corrosive	Neutral	High corrosive	Non aggressive
S38	Corrosive	Corrosive	Neutral	High corrosive	Mildly aggressive
S39	Scaling	Scaling	Scaling	High corrosive	non aggressive
S40	Scaling	Corrosive	Neutral	Corrosion may occur	Mildly aggressive
S41	Scaling	Neutral	Neutral	High corrosive	Non aggressive
S42	Corrosive	Corrosive	Corrosive	High corrosive	Mildly aggressive
S43	Corrosive	Corrosive	Corrosive	High corrosive	Mildly aggressive
S44	Scaling	Scaling	Scaling	High corrosive	Non aggressive
S45	Scaling	Neutral	Scaling	High corrosive	Non aggressive
S46	Scaling	Neutral	Scaling	Non corrosive	Non aggressive
S47	Scaling	Neutral	Scaling	High corrosive	Non aggressive
S48	Scaling	Scaling	Scaling	High corrosive	Non aggressive
S49	Scaling	Scaling	Scaling	High corrosive	Non aggressive
S50	Scaling	Neutral	Neutral	High corrosive	Non aggressive

## 2 Experimental design, materials, and methods

### 2.1 Study area description

The South West District of N.C.T. of Delhi is situated between latitude 28 40′ and 28 29′ and longitude between 76 50′ and 77 14′. The South West district has a varied character with Kapashera Sub Division as predominantly rural and the Dwarka Sub Division as mostly urban and Najafgarh Sub Divisions as a mix of both urban and rural population.

### 2.2 Sample collection and analytical procedures

The samples were collected in thoroughly cleaned 2 L capacity bottles and stored at a suitable temperature with necessary precautions till the analysis was done. All sampling sites were selected with a view to cover the entire area of study. Parameters such as pH, EC, SA, and TDS were measured in the field and crosschecked in the laboratory using water analysis kit (NPC 365, India). Other parameters such as TH, Ca^+2^, Mg^+2^ were measured by EDTA titrimetric method. Na^+^, K^+^ were measured using Flame photometer (Toshniwal TMF-45, India). Cl^−1^ contents were measured by ergonometric titration. F^−^ was determined using SPANDS method, The concentration of $N{O_{3}}^{-}$ and $SO_{4}^{-2}$ was determined using UV–vis Spectrophotometer (Hitachi U-2900, India) at wavelength 220 nm and 420 nm respectively.
