# Supplementary figures and images for: Assessment and hydro-geochemical characterization for evaluation of corrosion and scaling potential of groundwater in South West Delhi, India
Source: Data Brief. 2018 Mar 31;18:928–38. doi: 10.1016/j.dib.2018.03.120 (PMC5996502; doi:10.1016/j.dib.2018.03.120)

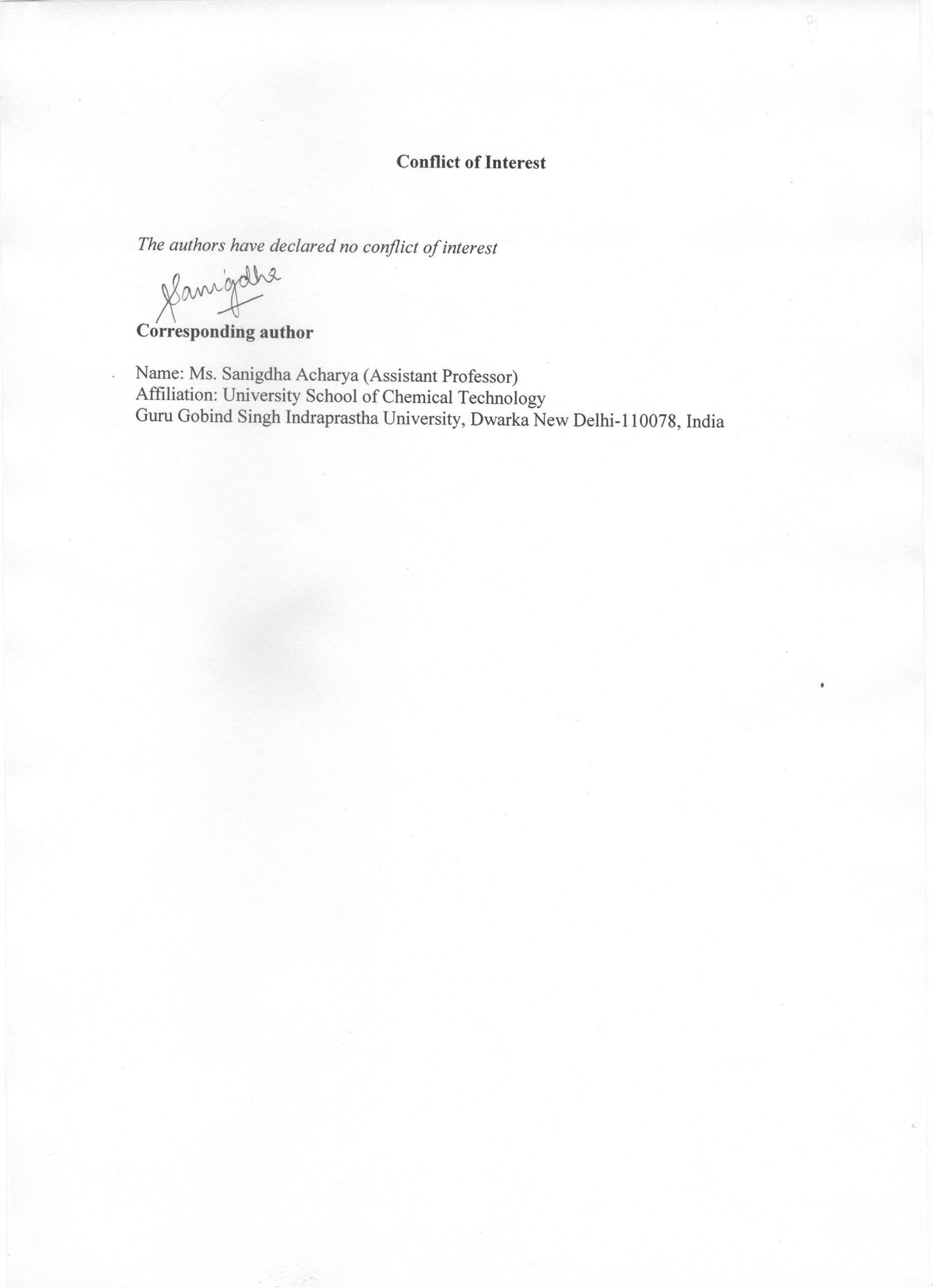

Supplement: Supplementary file 1 — Supplementary material [file mmc1.jpg]
